# Effects of Pre-Term Birth on the Cardio-Respiratory Responses to Hypoxic Exercise in Children

**DOI:** 10.3390/life12010079

**Published:** 2022-01-06

**Authors:** Benjamin J. Narang, Giorgio Manferdelli, Katja Kepic, Alexandros Sotiridis, Damjan Osredkar, Nicolas Bourdillon, Grégoire P. Millet, Tadej Debevec

**Affiliations:** 1 Department of Automatics, Biocybernetics and Robotics, Jožef Stefan Institute, 1000 Ljubljana, Slovenia; tadej.debevec@fsp.uni-lj.si; 2 Faculty of Sport, University of Ljubljana, 1000 Ljubljana, Slovenia; katja.kepic@gmail.com; 3 Institute of Sport Sciences, University of Lausanne, 1015 Lausanne, Switzerland; giorgio.manferdelli@unil.ch (G.M.); nicolas.bourdillon@unil.ch (N.B.); gregoire.millet@unil.ch (G.P.M.); 4 School of Physical Education and Sport Science, National and Kapodistrian University of Athens, 17237 Athens, Greece; asotiridis@phed.uoa.gr; 5 Department of Pediatric Neurology, University Children’s Hospital Ljubljana, 1000 Ljubljana, Slovenia; damjan.osredkar@kclj.si; 6 be.care SA, 1020 Renens, Switzerland

**Keywords:** altitude, children, exercise capacity, hypoxia, prematurity

## Abstract

Pre-term birth is associated with numerous cardio-respiratory sequelae in children. Whether these impairments impact the responses to exercise in normoxia or hypoxia remains to be established. Fourteen prematurely-born (PREM) (Mean ± SD; gestational age 29 ± 2 weeks; age 9.5 ± 0.3 years), and 15 full-term children (CONT) (gestational age 39 ± 1 weeks; age 9.7 ± 0.9 years), underwent incremental exercise tests to exhaustion in normoxia (FiO_2_ = 20.9%) and normobaric hypoxia (FiO_2_ = 13.2%) on a cycle ergometer. Cardio-respiratory variables were measured throughout. Peak power output was higher in normoxia than hypoxia (103 ± 17 vs. 77 ± 18 W; *p* < 0.001), with no difference between CONT and PREM (94 ± 23 vs. 86 ± 19 W; *p* = 0.154). VO_2_peak was higher in normoxia than hypoxia in CONT (50.8 ± 7.2 vs. 43.8 ± 9.9 mL·kg^−1^·min^−1^; *p* < 0.001) but not in PREM (48.1 ± 7.5 vs. 45.0 ± 6.8 mL·kg^−1^·min^−1^; *p* = 0.137; interaction *p* = 0.044). Higher peak heart rate (187 ± 11 vs. 180 ± 10 bpm; *p* = 0.005) and lower stroke volume (72 ± 13 vs. 77 ± 14 mL; *p* = 0.004) were observed in normoxia *versus* hypoxia in CONT, with no such differences in PREM (*p* = 0.218 and >0.999, respectively). In conclusion, premature birth does not appear to exacerbate the negative effect of hypoxia on exercise capacity in children. Further research is warranted to identify whether prematurity elicits a protective effect, and to clarify the potential underlying mechanisms.

## 1. Introduction

Each year an estimated 15 million newborns survive premature birth [1]. While a continual increase in survival rate reflects positive advancements in neonatal medicine, many survivors exhibit physiological impairments which appear to persist into adulthood [2]. Such impairments include a reduced lung diffusion capacity for carbon monoxide [3,4], a lower exercise capacity in normoxia [5], and a potentially altered ventilatory response to hypoxia [6,7].

The effects of pre-term birth on the physiological responses to various stimuli in children remain poorly investigated. Contrary to differences seen in adults [5], O’Dea et al. recently observed similar oxygen uptake (VO_2_peak), heart rate and minute ventilation (V_E_) at peak exercise intensity in children aged 9–12 years born pre-term (gestational age (GA) < 32 weeks), relative to their full-term counterparts, during an incremental treadmill test in normoxia [8]. These similarities were detected even in pre-term children diagnosed with bronchopulmonary dysplasia (BPD), despite the high prevalence of structural lung abnormalities among the pre-term group (88% of participants). In another study that examined extremely pre-term children (GA ≤ 28 weeks; participation age 8–12 years), a substantially lower VO_2_peak was observed in pre-term children with moderate-to-severe BPD relative to those with no-to-mild BPD and term-born children [9]. No significant differences were observed between these latter groups. Taken together, these studies suggest that the presence of BPD could be an important mediator determining the association between prematurity and exercise capacity. In addition, prematurely-born neonates who developed BPD may have reduced cardio–respiratory fitness through childhood due to a lower physical activity level. Further research is required to establish whether prematurity per se could influence the physiological responses to exercise in children.

While research investigating the cardio–respiratory effects of prematurity in children is growing, few studies have explored the responses to hypoxic exposure in this population. Given the increase in altitude sojourns for leisure purposes, it is important to establish whether children born pre-term are at increased risk of adverse responses. Joshi et al. (2014) observed an accentuated decrease in pulse oxygen saturation (SpO_2_) in children with a chronic lung disease of prematurity, relative to pre-term and full-term control groups, during exposure to a 12% oxygen stimulus at rest [10]. However, no differences were observed in the cardiovascular responses to hypoxia. Moreover, given that participants were studied at rest, the findings do not indicate the contribution of exercise as a concurrent stimulus. Indeed, we previously demonstrated a blunted resting hypoxic ventilatory response in pre-term relative to full-term adults, but this difference was negated during submaximal exercise [7]. Whether this differential response also exists in pre-term children remains to be investigated.

Accordingly, the aim of this study was to assess the cardio–respiratory responses to hypoxic exposure at rest and throughout an incremental exercise test in pre-term children, relative to their full-term counterparts. Based on previous research in adults [7], pre-term children were hypothesized to exhibit a blunted resting hypoxic ventilatory response. These differences were hypothesized to be attenuated during exercise. Moreover, pre-term children were hypothesized to have a reduced exercise capacity relative to full-term children. 

## 2. Materials and Methods

### 2.1. Study Design and Participants

This study adopted a case-control research design, whereby prematurely-born children (*n* = 14; PREM) were compared to age and activity-matched full-term controls (*n* = 15; CONT). This study was conducted at the University of Ljubljana, Slovenia, between November and December 2017. The pre-term born children were recruited by means of letter invitations to them and their parents based on the national pre-term birth register data available at the University Clinical Centre in Ljubljana, Slovenia. The full-term born children were invited to take part via web-based invitations/advertisements and personal contacts. The age (Mean ± SD) of the PREM and CONT groups at the time of testing was 9.5 ± 0.3 years and 9.7 ± 0.9 years, respectively (*p* = 0.386). Participant characteristics are displayed in Table 1. Participants were eligible for PREM if their GA < 32 weeks and their birth weight was <1500 g, while full-term participants were required to have a GA ≥ 38 weeks and a birth weight ≥ 2500 g. Participants were included if they had not been diagnosed with any respiratory, hematological or cardiac diseases, including BPD. The participants visited the laboratory for three tests, having had no exposure to altitude > 1500 m in the previous two months, and having abstained from exercise for 24 h prior to each visit. During the first visit, participants were pre-screened, familiarized with the protocols, and completed a baseline lung function test. Participants then performed incremental exercise tests to exhaustion on a cycle ergometer; one in normoxia and the other in normo-baric hypoxia in a randomized, counterbalanced order. Written informed consent was obtained from the children and their parents prior to study participation. The study was approved by the National Committee for Medical Ethics at the Ministry of Health of the Republic of Slovenia (0120-101/2016-2), and conducted in accordance with the Declaration of Helsinki.

### 2.2. Lung Function Test

During their first laboratory visit, participants completed a lung function test using a pneumotachograph (Cardiovit AT-2plus, Schiller, Baar, Switzerland) following standardized spirometry protocols [12]. Forced vital capacity, forced expiratory volume in the first second of exhalation, and peak expiratory flow rate were extracted from the resulting maximal flow volume loops. Predicted lung function was calculated using the Global Lung Function Initiative 2012 equations [11]. These values were determined according to the age, height and biological sex of each participant. The absolute and relative values for lung function are presented in Table 1.

### 2.3. Incremental Exercise Tests

Participants performed incremental exercise tests to exhaustion under normobaric normoxic (Mean ± SD; barometric pressure (BP), 739.9 ± 5.9 mmHg; inspired fraction of oxygen (FiO_2_), 20.9%; partial pressure of oxygen (PO_2_), 154 ± 1 mmHg) and normobaric hypoxic (BP, 740.2 ± 6.1 mmHg; FiO_2_, 13.24 ± 0.04%; PO_2_, 98 ± 1 mmHg) conditions in a single-blinded fashion. Tests were completed on an electromagnetically-braked cycle ergometer (Ergo Bike Premium, Daum Electronics, Fürth, Germany) with a targeted cadence of 60–80 rpm. Both conditions began with three minutes of seated rest in normoxia, after which participants completed a further three minutes of seated rest under hypoxic conditions in their hypoxia trial. Participants then began cycling at 0 W, with the workload increasing by 25 W every three minutes until volitional exhaustion. Verbal encouragement was provided by the researchers during the latter stages of both tests.

Participants breathed through an oro-nasal mask (VmaskTM, Hans Rudolph, Shawnee, KS, USA) via a two-way low-resistance valve (2700 NRBV; Hans Rudolph, Shawnee, KS, USA), connected to a 200-L Douglas bag containing either the normoxic or hypoxic gas mixture. Breath-by-breath gas exchange and ventilatory variables were monitored continuously using a metabolic cart (Quark CPET, Cosmed, Rome, Italy) which was calibrated before every test. SpO_2_ was continuously measured using a fingertip pulse oximeter (BCI 3301, Nellcor, Minneapolis, MN, USA). A hemodynamic monitor (PhysioFlow^®^, Manatec Biomedical, Poissy, France) was used to continuously record cardiac variables, and was set up according to the manufacturer’s guidelines.

### 2.4. Data Processing

Data processing was conducted in Microsoft Excel 2016 (Microsoft Corporation, Redmond, Washington, DC, USA). Breath-by-breath response profiles were smoothed using a five-point moving median to remove outlier breaths from the trace. The final 60-s average prior to the onset of exercise was extracted to determine resting values, and the 30 s prior to exhaustion were averaged to identify peak values. The arteriovenous oxygen difference was calculated as the average rate of oxygen uptake divided by average cardiac output [13]. Peak power output was determined as the power output at the highest completed stage, plus the proportion of the subsequent increment that reflected the duration of the final stage that the participant completed [14].

### 2.5. Statistical Analyses

All data are displayed as Mean ± SD. Statistical analyses were conducted in SPSS v27 (IBM Corporation, Armonk, New York, NY, USA). A priori sample size estimations were calculated using G*Power software (version 3.1.9.3) and data from previous research conducted in adults [7]. The lowest effect size (Cohen’s *d*) observed in the primary outcomes—the ventilatory response to hypoxia—was 1.18, so 13 participants per group were required for this study (*α* = 0.05; *β* = 0.18). Differences were assessed using a two-way mixed-effects ANOVA (group (PREM, CONT) * condition (hypoxia, normoxia)), irrespective of minor deviations from normality in the underlying data [15]. A Bonferroni post-hoc test was applied to investigate interaction effects in greater detail. Statistical significance in all cases was accepted at *p* < 0.05.

## 3. Results

### 3.1. Rest

Table 2 shows the cardio-respiratory variables at rest. A main effect of condition was observed in multiple variables, including VO_2_, V_E_, V_T_, Bf, SpO_2_, heart rate, stroke volume and cardiac output. A main effect of group was observed in cardiac output, such that PREM had a lower resting cardiac output than CONT. There were no main effects of group or group*condition interaction effects in any other variables.

### 3.2. Peak

**Figure 1:**
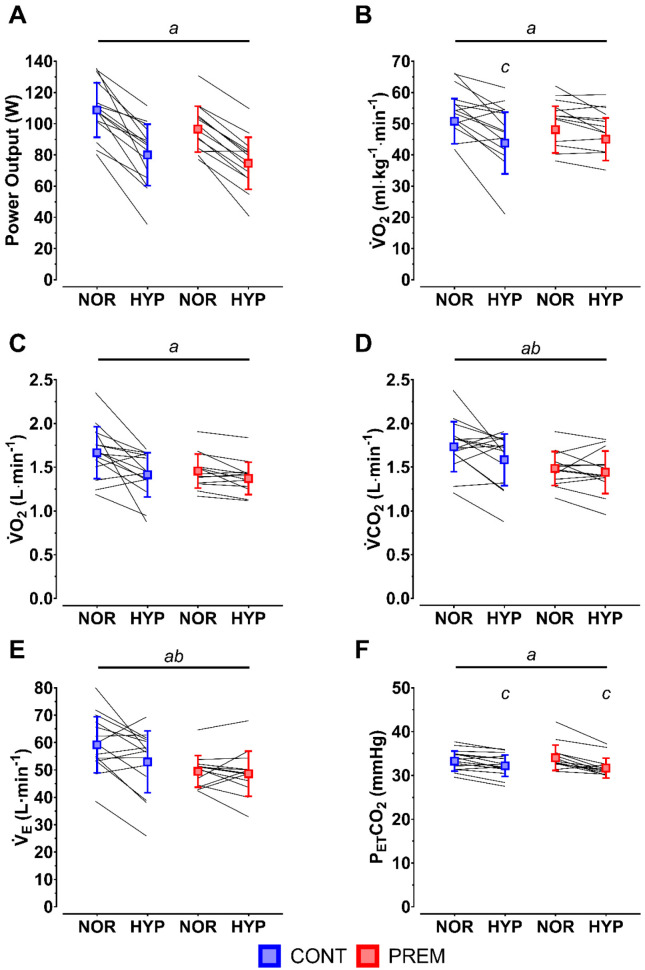
Power output (**A**), relative (VO_2_peak; (**B**)) and absolute (VO_2_; (**C**)) oxygen uptake, carbon dioxide production (VCO_2_; (**D**)), minute ventilation (V_E_; (**E**)) and end-tidal partial pressure of carbon dioxide (P_ET_CO_2_; (**F**)) in control (CONT; blue) and pre-term (PREM; red) children under normoxic (NOR) and hypoxic (HYP) conditions prior to exhaustion. Individual data superimposed as solid black lines. *^a^*main effect of condition; *^b^*main effect of group; *^c^*within-group difference vs. NOR.

Peak power output was higher in normoxia than hypoxia (*p* < 0.001), but there was no main effect of group (*p* = 0.154) or group*condition interaction (*p* = 0.148) (Figure 1A). Relative VO_2_peak was greater in normoxia than in hypoxia (*p* < 0.001) (Figure 1B). A significant interaction effect (*p* = 0.044) revealed that the difference in relative VO_2_peak between normoxia and hypoxia was significant in CONT (*p* < 0.001) but not in PREM (*p* = 0.137) (Figure 1B).

Peak absolute VO_2_ (*p* < 0.001) (Figure 1C), VCO_2_ (*p* = 0.043) (Figure 1D) and V_E_ (*p* = 0.027) (Figure 1E) were significantly lower in hypoxia than in normoxia. Main effects of group were also observed in VCO_2_ (*p* = 0.032) (Figure 1C) and V_E_ (*p* = 0.035) (Figure 1E). P_ET_O_2_ (normoxia vs. hypoxia; 113 ± 3 vs. 62 ± 4 mmHg; *p* < 0.001) and P_ET_CO_2_ (*p* < 0.001) (Figure 1F) were greater in normoxia than in hypoxia. The decrease in P_ET_CO_2_ from normoxia to hypoxia was greater in PREM (*p* < 0.001) than it was in CONT (*p* = 0.005; interaction *p* = 0.025) (Figure 1F).

**Figure 2:**
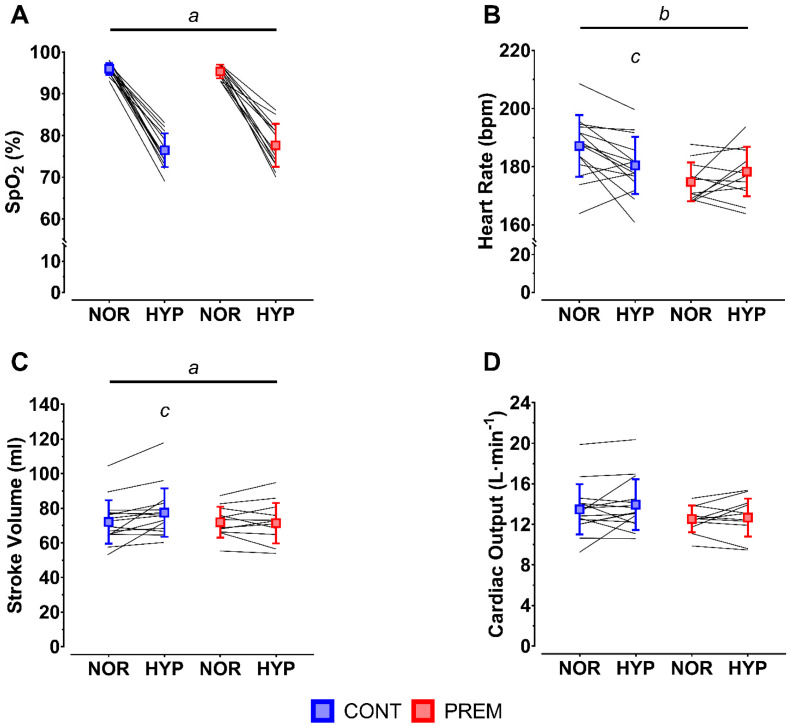
Capillary oxygen saturation (SpO_2_; (**A**)), heart rate (**B**), stroke volume (**C**) and cardiac output (**D**) in control (CONT; blue) and pre-term (PREM; red) children under normoxic (NOR) and hypoxic (HYP) conditions prior to exhaustion. Individual data superimposed as solid black lines. Note the broken y-axes in panels A and B. *^a^* main effect of condition; *^b^* main effect of group; *^c^* within-group difference vs. NOR.

SpO_2_ at exhaustion was lower in hypoxia than it was in normoxia (*p* < 0.001) (Figure 2A). Heart rate was significantly lower in PREM than in CONT (*p* = 0.027) (Figure 2B). A greater heart rate in normoxia than hypoxia was observed in CONT (*p* = 0.005), whereas heart rate was similar between conditions in PREM (*p* = 0.218; interaction *p* = 0.006) (Figure 2B). Stroke volume was significantly lower in normoxia than hypoxia (*p* = 0.047); a difference accounted for entirely by CONT (*p* = 0.004) rather than PREM (*p* > 0.999; interaction *p* = 0.047) (Figure 2C). No main effect of group (*p* = 0.220), main effect of condition (*p* = 0.194), or group*condition interaction (*p* = 0.750) was observed in cardiac output (Figure 2D).

## 4. Discussion

This study was designed to investigate the effects of acute hypoxia during incremental exercise in prematurely-born children. These were compared to normoxic conditions and children born full-term. VO_2_peak was reduced in hypoxia to a greater extent in CONT than in PREM, suggesting that premature birth does not accentuate the debilitative effect of hypoxia on exercise capacity. Pre-term children also displayed a lower peak V_E_ and heart rate. At rest, differences were primarily observed between conditions, although pre-term children displayed a reduced cardiac output relative to their full-term counterparts. Exercise capacity was reduced in hypoxia to a greater extent in CONT than in PREM. Pre-term children also displayed a lower peak V_E_ and heart rate. Regarding baseline characteristics, in accordance with previous research [3,16,17], the pre-term participants in this study demonstrated a reduced peak expiratory flow rate and relative forced expiratory volume in the first second of exhalation than the full-term control group. 

A key characteristic of premature birth is the altered ventilatory control that persists through maturity. For example, dysfunctional ventilatory responses to both hypoxic and hyperoxic stimuli have been observed in pre-term infants [18,19] and adults [6,7]. Conversely, the present study observed a similar hypoxia-induced increase in resting V_E_ between pre-term children and full-term controls, suggesting the acute hypoxic ventilatory response per se was not impaired by prematurity in this sample. This discrepancy may reflect that, in contrast to the previous research, BPD was an exclusion criterion in this study. As BPD per se is believed to be associated with impaired chemosensitivity [20], secondary to immature alveolar development [21], prematurely-born children without BPD may be less susceptible to an impaired hypoxic ventilatory response. When considering exercise as a stimulus for ventilatory changes, evidence also suggests a potentiated response in pre-term children with moderate-to-severe BPD relative to both pre-term children with no-to-mild BPD and full-term controls, with no difference between the latter two groups [9]. Further research is necessary to establish the independent impact of prematurity on the ventilatory responses to both exercise and hypoxia.

Exercise capacity was similar between prematurely-born children and their full-term counterparts under normoxic conditions. This is in line with previous research conducted in children [8,9], although an impaired exercise capacity with prematurity in normoxia has often been observed in adults [5,22]. A key novelty of this study was the translation of these comparisons to normobaric hypoxic conditions. The results suggest an attenuated reduction in relative VO_2_peak from normoxia to hypoxia in pre-term relative to full-term children. Similar findings have also been reported in adults. For example, a lower power output at volitional exhaustion was detected in pre-term individuals in normoxia compared to their full-term counterparts, but no between-group differences were detected in hypoxia [23]. Together, these findings indicate that premature birth may evoke a protective effect against the negative influence of hypoxia on exercise capacity. With the current state of the literature, this claim remains tentative. Further specifically-designed research is certainly necessary to explore the potential for prematurity-induced protection, and the mechanisms that could underpin this effect. Acute hypoxic exposure [24] and intense bouts of aerobic exercise [25] both induce oxidative stress, with the combined effect of both stimuli seemingly additive [26]. While pre-term adolescents have increased airway oxidative stress relative to full-term controls [16], the increase in exercise-induced oxidative stress with hypoxia was found to be attenuated in pre-term adults compared to aerobic capacity-matched full-term controls [27]. Therefore, although speculative without direct measurements, an enhanced ability to modulate hypoxic exercise-induced oxidative stress could explain the superior maintenance of exercise capacity in pre-term children. Whether these putative mechanisms can indeed be translated to a protective effect remains to be seen, but these studies certainly suggest that healthy prematurely-born individuals are able to tolerate hypoxic exercise at least as well as their full-term counterparts.

Regarding the cardiac parameters, heart rate was lower in pre-term relative to full-term children immediately before volitional exhaustion. However, there was no effect of prematurity on stroke volume or cardiac output. This contradicts previous research that found similar peak heart rates in pre-term and full-term children [8], although this discrepancy could be explained by differing exercise modalities. Treadmill running is known to elicit higher maximal heart rates than cycling [28], so it is plausible that heart rate limitations were not responsible for exhaustion in the present study. Regarding the hypoxic stimulus, a recent meta-analysis of 86 studies reporting the effects of acute hypoxia on maximal heart rate found a dose-response relationship, such that maximal heart rate decreased with increasing hypoxia [29]. However, high variability was found both within and between studies, and there is currently limited evidence in clinical populations. In this study, hypoxia induced a lower peak heart rate in control participants only, with a concomitant increase in stroke volume such that cardiac output was similar between conditions. In pre-term children, a comparable cardiac output between conditions was induced by similar heart rates and stroke volumes at peak exercise intensity. Although this suggests a prematurity-induced difference in the manifestation of peak cardiac output in hypoxia, the relevance of this finding is not immediately clear. It may provide additional evidence that pre-term individuals are affected to a lesser extent by hypoxia during exercise, further indicating that physical activity at altitude should not be discouraged in this population.

While this is the first study to investigate the effects of hypoxic exercise in prematurely-born children, some limitations warrant acknowledgment. First, the variability in several outcome measures was relatively high, so similarities between groups may be due to a lack of statistical power in those comparisons. However, our previous work in children cohorts [30,31] suggested that this study was sufficiently powered to detect differences in exercise capacity. Furthermore, participants with BPD were excluded from the study which, while allowing an investigation of prematurity per se, may underestimate the effect of pre-term birth in relation to the entire population. On the other hand, since moderate-to-severe BPD has been shown to reduce exercise capacity independently from pre-term birth [9], it is feasible that the inclusion of children diagnosed with BPD could reverse the interpretations surrounding the hypoxic stimulus. Future research that recruits prematurely-born participants with and without BPD, and compares their responses to full-term control participants, is certainly warranted to establish the independent and combined effects of BPD and prematurity. Finally, ventilatory parameters are notoriously difficult to study in children and erratic breathing patterns during exercise were indeed common in this sample. It is therefore possible that the low signal-to-noise ratio in the breath-by-breath response profiles of the participants might have reduced the validity of the variables associated with gas exchange. However, the error in each individual datapoint is likely to be random rather than systematic, and the use of a five-point moving median provided an unbiased approach to removing outliers and identifying a stable and reflective breathing pattern. In addition, the PhysioFlow device is yet to be validated against gold-standard methods for use in children, and may be biased towards overestimation due to the confounding effects of movement and artefacts induced by respiratory pattern [32]. Collectively, considering the challenges associated with these types of data, researchers are encouraged to comprehensively describe the strategies utilized to collect, process and ultimately analyze breath-by-breath and hemodynamic response profiles in children and/or under conditions of environmental stress.

## 5. Conclusions

The results of this study suggest that prematurely-born children have a lower exercise capacity, expressed as relative VO_2_peak, than their full-term counterparts under normoxic conditions. However, the debilitating effects of hypoxia on exercise capacity appear to be attenuated in pre-term children. This is a promising finding for those that wish to engage in physical activity at altitude. Further research is required to consolidate these findings and to identify the limiting factors to exercise capacity in this population.

## Figures and Tables

**Table 1:** Baseline participant characteristics for the control (*n* = 15) and the pre-term (*n* = 14) groups, and results of the lung function testing.

	Control	Pre-Term
Age at Test (years)	9.7 ± 0.9	9.5 ± 0.3
Body Mass (kg)	33.1 ± 7.1	30.9 ± 5.4
Height (m)	1.40 ± 0.07	1.39 ± 0.07
Body Mass Index (kg·m^−2^)	16.8 ± 2.5	15.8 ± 1.6
Body Fat Percentage (%)	17.7 ± 6.6	16.7 ± 6.0
Birth weight (g)	3270 ± 307	1202 ± 184 **
Gestational Age (weeks)	39.3 ± 1.3	29.3 ± 1.8 **
FVC (L)	2.11 ± 0.24	2.11 ± 0.42
FVC (% predicted)	93 ± 7	93 ± 10
FEV_1_ (L)	1.91 ± 0.25	1.82 ± 0.37
FEV_1_ (% predicted)	97 ± 9	93 ± 10
FEV_1_/FVC (%)	90.8 ± 4.1	86.4 ± 6.5 *
FEV_1_/FVC (% predicted)	103 ± 5	99 ± 7
PEF (L·min^−1^)	4.24 ± 0.95	3.46 ± 0.89 *

Note: Predicted lung function parameters calculated using the equations provided by Quanjer et al. (2012) [11]. Values are Mean ± SD. * *p* < 0.05; ** *p* < 0.01 vs. CONTROL. FEV_1_, forced expiratory volume in one second; FVC, forced vital capacity; PEF, peak expiratory flow rate.

**Table 2:** Selected cardio-respiratory variables at rest.

	Normoxia		Hypoxia		Anova (*p*-Value)		
	Control	Pre-Term	Control	Pre-Term	Main Effect Group	Main Effect Condition	Interaction Effect Condition*Group
VO_2_ (L·min^−1^)	0.32 ± 0.07	0.33 ± 0.04	0.36 ± 0.10	0.38 ± 0.06	0.463	**0.019**	0.706
VO_2_ (ml·kg^−1^·min^−1^)	9.7 ± 1.8	11.0 ± 2.5	11.2 ± 3.6	12.6 ± 3.2	0.188	**0.006**	0.844
VO_2_ (%VO_2_peak)	20.3 ± 5.7	22.9 ± 3.8	25.0 ± 6.5	28.3 ± 7.2	0.104	**0.001**	0.832
VCO_2_ (L·min^−1^)	0.29 ± 0.06	0.30 ± 0.05	0.38 ± 0.09	0.40 ± 0.06	0.498	**<0.001**	0.660
RQ	0.92 ± 0.07	0.91 ± 0.06	1.13 ± 0.22	1.08 ± 0.07	0.333	**<0.001**	0.591
V_E_ (L·min^−1^)	9.3 ± 2.0	9.6 ± 1.4	12.1 ± 2.8	13.0 ± 2.0	0.411	**<0.001**	0.571
V_E_ (%V_E_peak)	16 ± 5	20 ± 3	24 ± 8	28 ± 7	0.095	**<0.001**	0.950
V_T_ (L)	0.47 ± 0.10	0.50 ± 0.13	0.57 ± 0.18	0.64 ± 0.12	0.292	**<0.001**	0.503
Bf (b·min^−1^)	20.3 ± 4.5	20.2 ± 3.4	23.1 ± 5.4	21.2 ± 3.7	0.432	**0.022**	0.341
SpO_2_ (%)	97.6 ± 0.7	97.1 ± 1.2	88.5 ± 2.9	90.2 ± 4.0	0.431	**<0.001**	0.126
HR (bpm)	97 ± 8	92 ± 11	107 ± 8	106 ± 13	0.355	**<0.001**	0.394
HR (%HRpeak)	52 ± 2	51 ± 4	60 ± 5	59 ± 7	0.437	**<0.001**	0.521
SV (ml)	55 ± 9	53 ± 8	62 ± 8	56 ± 7	0.178	**<0.001**	0.070
CO (L·min^−1^)	5.4 ± 0.9	4.9 ± 0.7	6.6 ± 0.9	5.8 ± 0.9	**0.040**	**<0.001**	0.380
P_ET_O_2_ (mmHg)	108 ± 4	110 ± 3	61 ± 4	61 ± 2	0.720	**<0.001**	0.337
P_ET_CO_2_ (mmHg)	33 ± 2	33 ± 1	33 ± 2	33 ± 1	0.649	0.358	0.825
EqO_2_	30.8 ± 4.1	30.1 ± 2.7	37.2 ± 8.4	34.8 ± 2.7	0.272	**<0.001**	0.585
EqCO_2_	32.7 ± 2.4	32.8 ± 1.7	32.6 ± 2.3	32.3 ± 1.6	0.463	**0.019**	0.706
A-V O_2_ diff (%)	5.9 ± 0.8	6.7 ± 1.2	5.4 ± 1.6	6.6 ± 1.7	0.188	**0.006**	0.844

Note: Values are Mean ± SD. Statistical results are from a two-way mixed-effects ANOVA (group (PREM, CONT), condition (hypoxia, normoxia)). Significant effects are displayed in bold for clarity. A-V O_2_ diff, arteriovenous oxygen difference; Bf, breathing frequency; CO, cardiac output; EqO_2_, ventilatory equivalent for oxygen; EqCO_2_, ventilatory equivalent for carbon dioxide; HR, heart rate; P_ET_CO_2_, end tidal partial pressure of carbon dioxide; P_ET_O_2_, end tidal partial pressure of oxygen; RQ, respiratory quotient; SpO_2_, pulse oxygen saturation; SV, stroke volume; VCO_2_, carbon dioxide production; V_E_, minute ventilation; VO_2_, oxygen uptake; V_T_ tidal volume.

## Data Availability

The data presented in this study are available on request from the corresponding author.
